# Development and Characterization of Niosomal Gel System using *Lallementia royaleana Benth.* mucilage for the treatment of Rheumatoid Arthritis

**DOI:** 10.22037/ijpr.2020.112887.14003

**Published:** 2020

**Authors:** Snigdha Bhardwaj, Sonam Bhatia

**Affiliations:** a *Meerut Institute of Engineering and Technology, Meerut (250005), Uttar Pradesh, India *; b *Department of Pharmaceutical Science, Shalom Institute of Health and Allied Sciences, SHUATS, Prayagraj (211007), Uttar Pradesh, India.*

**Keywords:** Bilayer vesicles, niosomal gels, Ibuprofen, Rheumatoid arthritis, phase microscopy, Lallemantia royaleana Benth. mucilage

## Abstract

Niosomes structural framework comprises of non-ionic surfactant-based microscopic lamellar structures which carries the potential to sustain the effect of drug from its delivery system. In present work, the attempt was made to identify the effect of different ingredients such as effect of Tweens and natural mucilage of *Lallemantia royaleana Benth.* on the performance of developed niosomal gel formulations in order to prolong the duration of action of drug and to minimize its side effects of topical conventional drug administration. All Ibuprofen loaded niosomes formulationswere prepared by ether injection method; using cetosteryl alcohol with different variants of Tweens and Spans. Various evaluation parameters were performed to confirm niosome formation. Further, the niosomes were incorporated into gel system and evaluated for i*n-vitro* permeability study (*ex-vivo*) on excised rat skin by membrane diffusion method and *in-vivo *study by carrageenan induced rat paw edema model. The best selected niosome formulation F9 gave no sedimentation, layer separation and unchanged particle shapes and thus selected for gel preparation using *Lallemantia royaleana Benth. *mucilage and carbopol in different ratios. *Ex-vivo* and i*n-vivo *studies indicated high skin retention and penetration rates within the skin for tests niosomal gel formulations (G1 & G2). The present study suggested that developed topical gel formulation provides enhance permeability and longer duration of drug action over conventional gels.

## Introduction

Rheumatoid arthritis is a systemic, chronic, long-term autoimmune disorder that leads to inflammation of the synovial joints, and surrounding tissues gradually leading to joint destruction. The pattern of joints affected is usually symmetrical which affects the small joints of the hand and feet. Activated macrophages are responsible for the production of inflammatory cytokines which results in inflammation, swelling, bone erosion leading to chronic pain, stiffness, and functional impairment. Non-steroidal anti-inflammatory drugs (NSAIDs) are one of the leading pain relief drugs used today. Their uses range from analgesic to antipyretic and can also have anti-inflammatory benefits. NSAIDs acts by cyclooxygenase enzyme inhibition resulting in anti-inflammatory action, the main factor limiting the oral use of NSAIDs is the development of gastrointestinal (GI) adverse events, ranging from dyspepsia to serious life-threatening events (1, 2).

Ibuprofen (2-(4-isobutylphenyl) propanoic acid), p*K*a 4.8, is a non-steroidal, anti-inflammatory drug with analgesic and antipyretic properties used for the treatment of osteoarthritis, rheumatoid arthritis, ankylosing spondylitis, etc. Ibuprofen has a mechanism of action, which includes inhibiting cyclooxygenase systems (COX-1 and COX-2) from synthesizing prostaglandins. Prostaglandins are responsible for the inflammation response that results within tissue in the body when infection or injury has occurred (3). The main disadvantages of this family of drugs include a relatively short plasma half-life (about 1-3 h) and dosing frequency of more than once a day makes it difficult to maintain the desired concentration of drug levels. It is also known to produce significant gut (gastric ulcer, gastrointestinal bleeding, and hemorrhage) and nephrotoxicity in humans under prolonged consumption. Therefore, the development of a drug delivery system that enables the controlled release of Ibuprofen could be highly beneficial, particularly in high dose-dependent treatments, as in the treatment of chronic diseases such as rheumatoid arthritis (4, 5).

Topical drug delivery means the application of a drug to skin for localized effects. Dermal drug delivery has several advantages like longer duration of action, dosing flexibility, reduced side effects, uniform plasma levels, and high patient compliance. For the permeation of drugs to this barrier, many technologies and systems have been investigated and one of the most promising techniques is the vesicular carrier for drug delivery through the skin (6, 7). 

In recent years non-ionic surfactant vehicles received great attention as potential drug delivery systems for different routes of administration. Several scientific literatures support the effectiveness of niosomal drug delivery for topical application. In the 1970s and 1980s, L’Oreal developed and patented niosomes. But, Lancome, the first product containing “niosome,” was introduced in 1987 by L’Oreal (1989) (8). Patel *et al.* (2012) develop niosomal gel as a transdermal nanocarrier for improved systemic availability of Lopinavir (9). On the similar lines, Asthana *et al.* (2016) investigated the delivery potential of topical niosomal gel loaded with Eetodolac, a drug used in osteoarthritis (10). Zubairu *et al.* (2015) developed the niosomal formulation of Gatifloxain coated with chitosan polymer which was found to be effective in transcorneal delivery of anti-infective agent (11). Ramkanth *et al.* (2018) investigated the potential of proniosomes as a transdermal drug delivery system for Atenolol (12). On the contrary, Piplani *et al.* (2015), develop niosome using 4-hydroxybenzaldehyde and Isovanillin to make ophthalmic carriers for topical ocular treatment (13). Kamboj *et al.* (2013) prepared Mefenamic acid loaded niosomal gel by thin-film hydration technique using different surfactants and the *in-vivo *study showed good inhibition of inflammation by the gel prepared with niosomes (14). Srikanth *et al.* (2010) prepared Meloxicam entrapped niosomes by thin-film hydration and the studies demonstrated that niosomal gel showed better pharmacological activity than the conventional meloxicam gel. Other than this, niosomes have also found significant application in the field of antitumor drug delivery, anti-diabetic therapy, and also well-established role diagnostics and imaging (15). 

Niosomes structural framework comprises of non-ionic surfactant-based microscopic lamellar structures that formed on the admixture of a non-ionic surfactant, cholesterol and diethyl ether with subsequent hydration in aqueous media. Due to the incorporation of non-ionic surfactants, niosomes have more penetrating properties; they acquired spherical shape (16-18). 

The characteristic properties of niosomes can be varied by changing vesicle composition, size, surface charged, lamiellarity and concentration. Several forces act inside the bilayer structure in respect to surfactant molecules such as van der Waals among surfactant molecules, repulsive forces among a charged group of surfactant molecules due to electrostatic interactions, entropic repulsive forces of head groups of surfactant molecule, short-acting repulsive forces, *etc.* are responsible for the vesicular structure of niosomes. The stability of niosomes depends on various factors such as the nature of drug encapsulated, type of non-ionic surfactant used, storage temperature, nature of lipids used, and inclusion of charged molecule. Niosomes can accommodate drug molecules with varying solubility due to the presence of hydrophilic, amphiphilic and lipophilic moieties in the structure. Thus, the vesicles may act as a drug reservoir or depot that can adjust the drug release and affinity for the target site. (19, 20). Niosomes offer several advantages over liposomes as higher chemical stability, enhanced skin penetration properties, and lower costs. Phospholipids are chemically unstable because of their predisposition to oxidative degradation and require special storage conditions and handling. Niosomes explore great potential as a drug delivery system due to controlled delivery of drug at a particular site, delayed clearance from the circulation protecting the drug from biological environment and restricting effects to target cells. Moreover, niosomes surfactants are biodegradable, biocompatible, and non-immunogenic (21-23). Niosomes for topical applications may increase the residence time of drug candidates in the stratum corneum and epidermis while minimizing the systemic absorption of drugs (24). 

*Lallemantia royaleana Benth*., commonly known as Balangu or Tukhme balanga, is an annual or biennial herb, belongs to family *Lamiaceae*. It is cultivated throughout Western Asia, Pakistan, the Northern region of India and different regions of Iran and Iraq (25-27). In the Unani system of medicines, it is believed that the plant hasvarious medicinal properties such as diuretic, cardiotonic, sedative, *etc*. Traditional observations reported that the plant and its parts retain the potential to cure various disease conditions such as common cold, fever, joint pain, rheumatism, renal disorder, weakness of heart, psychotic disorder, and infections (28-31).

Seeds of *Lallemantia royaleana Benth.* are smooth textured and dark-brown to black. The chemical analysis of seeds of *Lallemantia royaleana Benth*. showed the presence of dry matter (92.75%), crude protein (25.60%), crude fat (18.27%), ash (3.63%), crude fiber (1.29%), alkaloids, anthraquinones, flavonoids, glycosides, tannins, volatile oils, mixed fatty acids, and terpenoids (32). The seeds develop translucent and voluminous mucilage coating when soaked in water. The mucilage is composed of L-rhamnose, D-galactose, L-arabinose, protein, uronic anhydride. It is generally used to treat inflammation, abscesses, and respiratory problems. The taste of mucilage is bland, soothing, and spicy (33, 34). Mucilage isolated from seeds exhibited excellent swelling property and also non-irritant to mucosal membrane. Many studies reported its use as a thickening agent, suspending agent, or super-disintegrating agent in formulations (35). In a study, mucilage of *Lallemantia royaleana Benth*. was tested as a gel supplement and natural matrix for sustained release of drugs. The results exhibited significant results in terms of potency and duration for test gel formulation (with natural mucilage) as compared to marketed gel preparation (36). Its mucilage can be used in the food and drink industry, such as ice cream and natural gel making (37). Several reports have been published on the analysis of seeds of *Lallemantia* species including *Lallemantia royaleana Benth*. The species consists of similar biologically active compounds such as polysaccharides, fixed oils, protein, soluble fibers, mucilages, and essential oils (38, 39).

The present study was carried out in an attempt to investigate the potential of Ibuprofen loaded niosomes gel formulation for the treatment of inflammatory conditions. Ibuprofen entrapped niosome formulations were developed by ether injection method and the prepared niosomes were further incorporated in the gel prepared with natural mucilage. Niosomes are said to have a sustained-release effect and better drug permeability. Niosomal gel can resist the physiological stress caused by skin flexion and thereby providing controlling drug release to the targeted site. The prepared topical niosomal gel would be highly beneficial to achieve better skin penetration, reducing the rheumatic inflammation as the required dose is decreased, and also circumvent the side effect of topical conventional drug administration (10). 

## Experimental

*Materials*

Ibuprofen and Cetosteryl alcohol were gifted by Medicamen Organics Limited, Sidcul, Uttrakhand, India. Carbopol 934, glycerine, propylene glycol, triethanolamine, benzalkonium chloride, Sorbitanmonostearate (Span 80), Sorbitanmonopalmitate (Span 40), Sorbitanmonolaurate (Span 20), Polysorbate 60 (Tween 60), Polysorbate 80 (Tween 80), Diethyl ether and methanol were provided by the institution research institute (SIHAS, SHUATS, Prayagraj, U.P., India). All the other reagents and solvents used were of analytical grade. The seed sample was purchased from the local market of Meerut City, Uttar Pradesh, India. A voucher herbarium specimen was prepared for necessary identification. *Lallemantia royaleana Benth. *plant and seeds authentication was done at the National Bureau of Plant and Genetic Resources, PUSA Campus, New Delhi, India. The voucher specimen was deposited to the Department of Pharmaceutics, M.I.E.T., Meerut, U.P., India for future evidence.

*Methods*

*Preparation of mucilage from Balangu seeds*

The process for mucilage extraction from seeds of *Lallemantia royaleana Benth*. was developed to separate swollen, translucent mucilaginous layer to collect non-starch part of seeds without crushing the seed core (which contains starch). Primarily seeds (200 g) were soaked in water (500 mL) overnight. After soaking, swelling of seeds occurred (dark grayish, thick translucent mucilaginous shell overseed core). The slow addition of cold water with concurrent stirring for 1 h using mechanical agitator led to loosening mucilage cover followed by mucilage separation from seeds. After agitation, the mixture (seed core and mucilaginous material) was allowed to pass through a muslin cloth to remove hard seed core from the pure mucilage. After the collection of pure mucilage, the washing was done with acetone in the ratio of 2:1 (Acetone: mucilage). After washing, the mucilage was dried at 35-40 °C. After drying, the mucilage was then ground to very fine powder followed by preservation at room temperature in airtight containers and wrapped up in aluminum foil to avoid contact with light. 

*Preparation of Ibuprofen loaded niosomes*

Niosomes were prepared by the ether injection method using non-ionic surfactants and cholesterol in different ratios, as stated in Table 1. The drug concentration was kept constant in all the formulations. The prepared niosomes were separated by ultracentrifugation (Remi C-24, Mumbai, India) at 4 °C (40, 41).

*Evaluations of Niosomes*

*Optical Microscopy and Phase contrast study*

Mean vesicle size analysis of the different formulations of Ibuprofen niosomes were measured by an optical microscope. In this analysis, an optical combination of the 10 X eye-piece lens and 10X objective lens was used. The measurements of different formulations of microspheres were done in triplicate and volume mean diameter (Vd) and standard deviation were recorded (42, 43). Besides, Phase contrast study was conducted to obtain topographical characteristics of vesicles, especially the shape and surface morphology. Different Ibuprofen loaded niosomal suspensions were deposited onto the separate glass slide and fixation was done by using a drop of glycerin. The glass slides were individually mounted on the phase contrast instrument and photographs were taken using phase contrast (Olympus Model BX 41, Japan).

*Estimation of percent yield values*

The total amounts of niosomes obtained were weighed by using a digital weighing balance (Citizen, Model CX 65, India). The percent yield was calculated by the following Equation (1):

Yield% = Amount of Niosomes (Drug + Cetosteryl alcohol + Surfactants) × 100 

 (Equation 1)

*Estimation of drug entrapment efficiency (DEE)*

The actual amount of Ibuprofen present in the different formulations of niosomes was estimated by ultra-centrifugation of niosomal suspension at 10,000 rpm for 1 h using at 4 °C. The amount of entrapped Ibuprofen was determined by lysis of the separated vesicles with Triton X-100. Thereafter, the filtered liquid was taken for the determination of Ibuprofen content spectrophotometrically by using a UV-VIS spectrophotometer (Shimadzu, model UV-1601 PC, Kyoto, Japan) at a wavelength of 221 nm against appropriate blank (44-46). The percent DEE was calculated by the following Equation 2: 

DEE% = (Actual drug content/Theoretical drug content) × 100

(Equation 2)

*Scanning electron microscope (SEM) analysis*

SEM images were taken for Tween 80 based Ibuprofen loaded niosomes (F_9_). Scanning was performed using a scanning electron microscope (LEO 435VP model, Cambridge, UK). The working distance of 26 mm was maintained and the acceleration voltage used was 15 kV with the secondary electron image (SEI) as a detector (47).

*ZETA Potential study*

The charges on Ibuprofen loaded vesicular surface were determined using Zetasizer DelsaTM Nano (Beckman Coulter, version 2.21) employing the phase analysis light scattering technique, which measures the particle electrophoretic mobility in a thermostated cell. The sample was analyzed 24 h after their preparation with an analysis time of 60 sec. The average zeta potential and charges were determined. The time-dependent correlation function on the scattered light intensity was measured at a scattering angle of 90°. 

*In-vitro drug release study*

*In-vitro* release studies of Ibuprofen from niosomal formulations were determined by the “Membrane Diffusion” method. An amount equivalent of Ibuprofen was placed in a glass tube (diameter: 2.5 cm and length: 8 cm), covered with soaked osmosis cellulose membrane, that was placed in a beaker containing 50 mL of phosphate buffer (pH 5.8), which acted as receptor compartment. The temperature of the receptor medium was maintained at 37 ± 1 °C and agitated at 100 rpm speed using a magnetic stirrer. Aliquots of 2 mL samples were withdrawn periodically and sink conditions were maintained. The collected samples were analyzed at 221 nm in Double beam UV-VIS spectrophotometer using phosphate buffer (pH 5.8) as blank. The cumulative percent release (CPR) up to 8 hours was calculated for all niosomal formulations (48, 49).

*Release Kinetics of Ibuprofen Niosomes*

To investigate the mode of drug release from Ibuprofen loaded niosomes, the release data were fitted with the following mathematical models (50-52). 

Zero-order kinetics Equation 3:

Qt = k0.t 

(Equation 3)

Where Qt is the amount of drug released at time t, k_0_ is the zero-order release rate constant, t is the time.

First-order kinetics Equation 4:

In Qt = In Q0 - k1.t 

(Equation 4)

Where, Qt is the amount of drug released at time t, Q_0_ is the initial amount of drug in the solution, k_1_ is the first-order release rate constant.

Higuchi model kinetics Equation 5:

Qt = kH.t1/2 

(Equation 5)

Where Qt is the amount of drug released at time t, k_H_ is the Higuchi release rate constant.

 Korsmeyer-Peppas model kinetics Equation 6:

Mt/M = KKP.tn 

(Equation 6)

Where Mt is the fraction of drug released at time t, M∞ is the fraction of drug released at infinite time, K_KP_ is the Korsmeyer-Peppas release rate constant, n is the release exponent.

Hixson-Crowell model kinetics Equation 7:

Q01/3 – Qt1/3 = KHC.t 

(Equation 7)

Where Q_0_ is the initial amount of the drug in the dosage form, Qt is the remaining amount of drug in the dosage form at time t, K_HC_ is the Hixson-Crowell release rate constant.

*Formulation Process*

*Percent yield of mucilage from Balangu seeds*

The percent yield of mucilage extracted from seeds of *Lallemantia royaleana Benth*. was found to be 8.67% w/w. Furthermore, the mucilage amount (%) used to develop niosomal gel was kept 1% and 1.5%.

*Preparation of niosomal gel*

The prepared niosomes were separated from aqueous medium by ultracentrifugation at 10,000 rpm at 40 °C and were gently added with a glass rod in the blank gel prepared by Carbopol-934 and *Lallemantia royaleana Benth*. mucilage in different ratios. The composition of gel is given in Table 2.

*Evaluation of Niosomes Loaded Gel*

*Ex-vivo diffusion studies*

Franz Diffusion cell was used to performed *ex-vivo* skin permeation studies for all Ibuprofen entrapped niosomal gel formulations (53). The receptor compartment contained phosphate buffer (pH 5.8) which was constantly stirred at 100 rpm with a small magnetic stirrer and controlled temperature at 37 ± 2 °C throughout the experiment. The abdominal skin of male rats (weighing 200 g) was taken for this study. The shaved rat skin was mounted with the stratum corneum side facing upwards to the donor compartment, and the subcutaneous side was in contact with the receiver medium. The gel was placed into the donor compartment and covered with paraffin film. The sample aliquots from the receiver chamber were collected at 30 min 1, 2, 3, 4, 5, 6, 7, and 8 h respectively and analyzed by UV-VIS Spectroscopy at 221nm. The amount of drug permeated, drug flux, and the permeation coefficients were calculated for niosomes loaded gel formulations (54, 55). 

*In-vivo anti-inflammatory study*

The animal study has been conducted as per the Animals (Scientific Procedures) Act. All the animals used in research work were cared for by trained, accountable staff, and housed in proper facilities. Before the animal study, the animal approval was obtained by IAEC (CPCSEA No. 711/02/a/CPCSEA) Anti-inflammatory study was performed by “Carrageenan induced rat paw edema” model using 24 albino rats of either sex weighing (100-150 g) and divided into 4 groups [shown in Table 3 (56). In all groups, acute inflammation was induced by a sub-planter injection of 0.1 mL of freshly prepared 1% suspension of carrageenan in normal saline in the left hind paw of the rats. The paw edema volume was measured using “Plethysmometer” at every 15 min interval for 2 h after the injection of carrageenan. The average paw edema volume of all the groups were calculated and compared with that of control (57). 

The percent inhibition of edema was calculated by using the following Equation 8:

Edema inhibition% = 1 – VtVc × 100                    

(Equation 8)

Where, Vt = Mean edema volume of test, Vc = Mean edema volume of control.

*Skin irritancy study*

The skin irritation test was performed on the healthy albino rat (200 g) for the best formulation by applying niosomes loaded gel formulation on the shaved portion of rat skin. The test was performed primarily by examining the rat to notice any changes after the application of the formulation. Then photographic imaging of an exposed portion of rat skin was taken out before and after subsequent application for 72 h that is after the study period and these images were compared determining the difference with the images taken before applying the formulation (58, 59). 

## Results

*Evaluation Results of Ibuprofen entrapped niosomal formulations*

In the present work total, ten niosomes formulations were prepared. The developed formulations were characterized for mean vesicle size; percent yield values, and entrapment efficiencies and the *in-vitro* drug release profile. The experiments were performed in triplicate to minimize error.

*Optical Microscopy and Phase contrast study*

**Figure 1:**
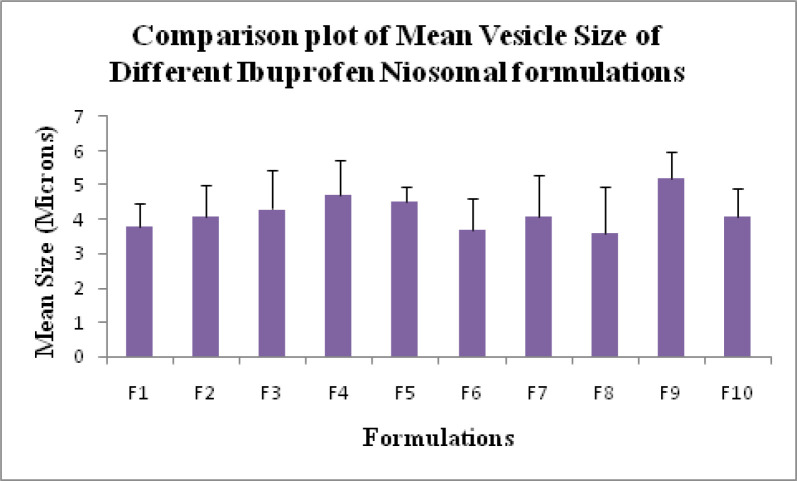
Mean Vesicle Size of all Ibuprofen loaded niosomes formulations (F1- F10).

**Figure 2:**
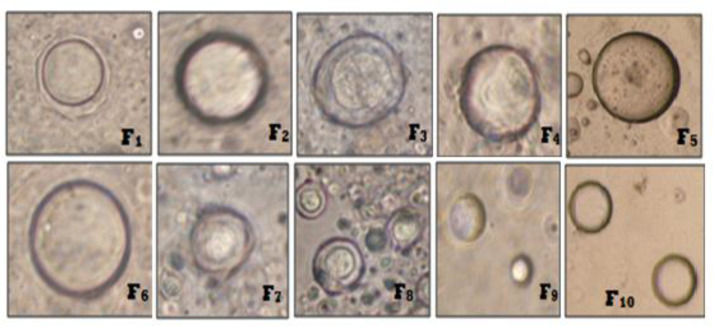
Phase Contrast Photomicrographs of Ibuprofen loaded niosomes formulations (F1-F10).

Mean vesicle size for all niosome formulations were measured using optical microscopy (19). Mean vesicle size data is shown in Table 1 and the comparative graphs of all niosome preparations for size using optical microscopy in Figure 1. From the Phase-contrast photomicrographs (100X magnification) of Ibuprofen entrapped niosomal formulation, the shape of niosomal vesicles was assumed to be spherical. Also, it was observed that formulation F9 has larger vesicle size but all the formulations have sphericity except F7 and F8 formulations. The phase-contrast photomicrographs of all niosome formulations are shown in Figure 2.  

*Percent Yield*

The percent yield values of all niosomal formulations were determined in triplicate along with mean values. The percent yield results for all batches of niosome formulations were found in the range of 53.6 ± 1.59 to 75.0 ± 0.87%. Formulation F9 has a good percent yield point (75.0 ± 0.87).

*Drug Entrapment efficiency*

**Figure 3:**
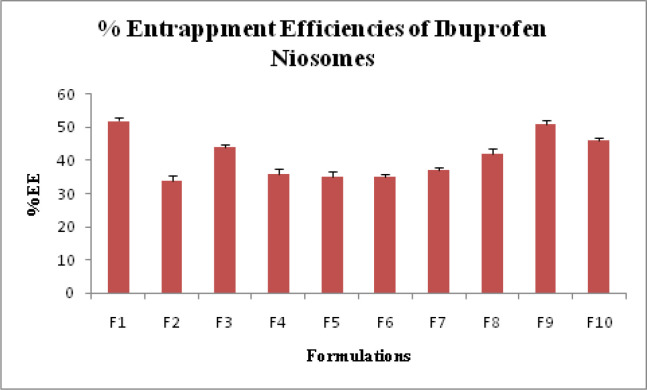
Entrapment Efficiency % of all Ibuprofen Loaded niosomes Formulation (F1- F10).

The percentage drug entrapment efficiency (DEE%) was determined in triplicate with their standard deviation (SD) values. DEE% of Ibuprofen loaded niosomes prepared from Span 20, Span 40, Span 60, Tween 60 and Tween 80 was found to be in the range of 34.0 ± 1.53% to 52.0 ± 1.0% and shown in Table 1 and Figure 3.

*Scanning electron microscope (SEM) analysis*

**Figure 4:**
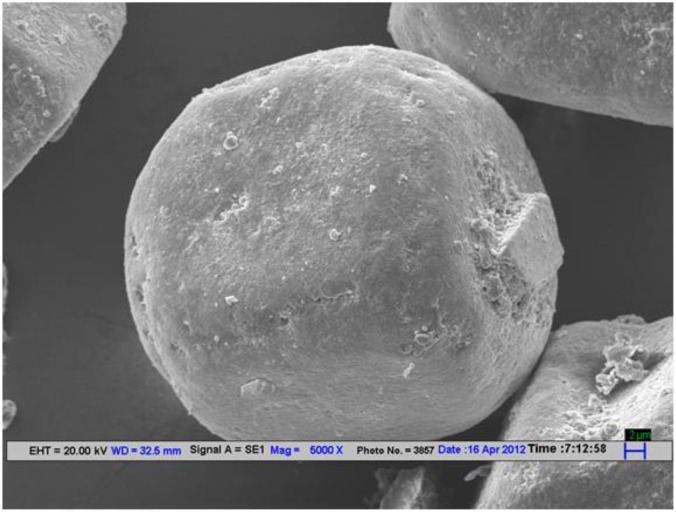
SEM image representing surface morphology of Ibuprofen loaded niosome formulation (F9).

 SEM micrographs of Ibuprofen loaded niosomes formulation F9 taken at different magnification were shown in Figure 4. SEM micrographs of a group and single of Ibuprofen loaded niosomes formulation (F9) were taken at 5000X magnification. 

*Zeta Potential Analysis*

**Figure 5:**
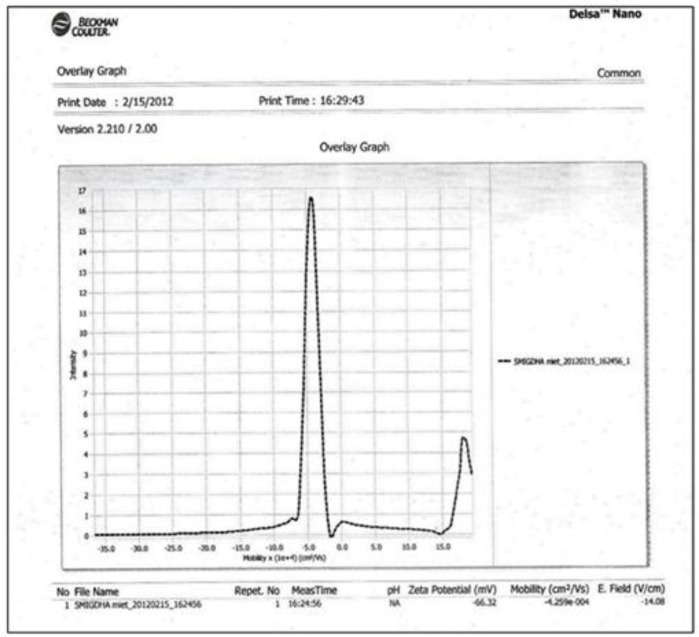
Zeta Potential Graph of Ibuprofen loaded Niosome formulation (F9).

ZETA Potential analysis was performed for Tween 80 based Ibuprofen loaded niosomes formulation (F9). The ZETA Potential value for this formulation was found to be -66.32 mV which indicated that the formulation was quite stable (Figure 5). 

*In-vitro drug release*

**Figure 6:**
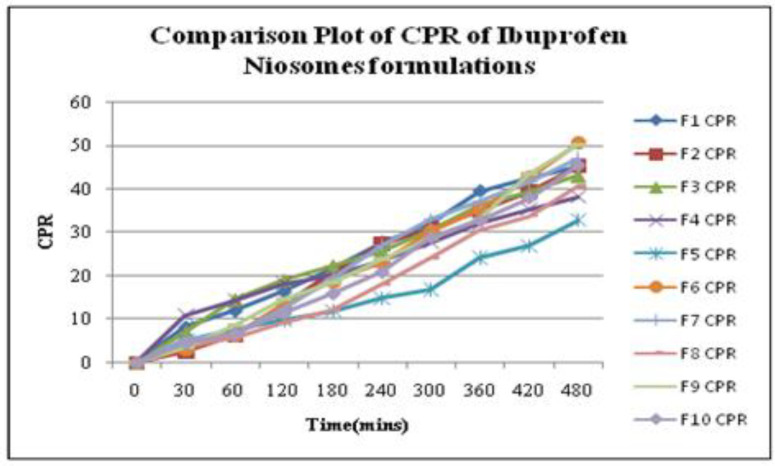
Comparative Plot representing Cumulative Percent Release (CPR) by all Ibuprofen loaded niosome formulations (F1-F10).

The *in-vitro* release profiles for each Ibuprofen entrapped niosome formulations in a phosphate buffer solution of pH 5.8 were determined in triplicate. The cumulative percent release (CPR) along with standard deviation for all niosomal formulation up to 8 h was calculated. The CPR data with a standard mean of all Ibuprofen entrapped niosomal formulations is given in Table1 and a comparative plot of cumulative release profiles for all niosome formulations is shown in Figure 6. Based on CPR profiles, the data of all niosome formulations can be arranged in the following decreasing order: F6 > F9 > F7 > F10 > F1 > F2 > F3 >F8 >F4> F5.

*Results of drug release kinetics*

**Figure 7:**
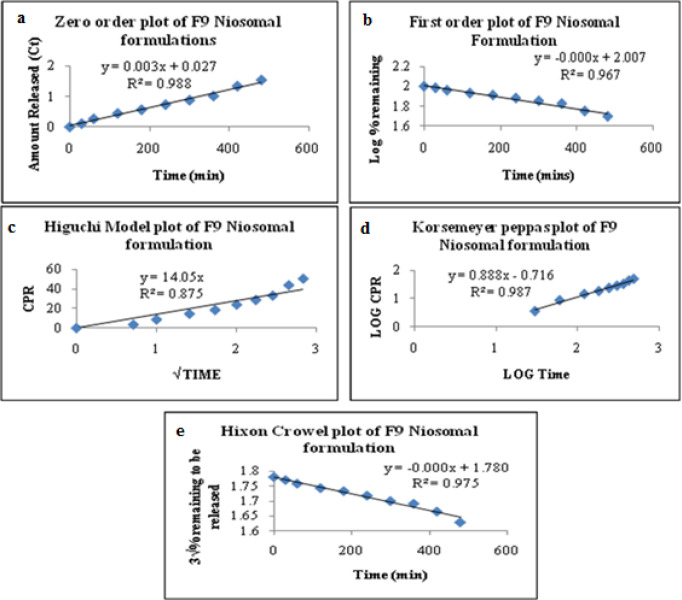
Release Kinetics representing Zero-order, First-order, Higuchi, Korsemeyer-Peppas and Hixon Crowel plots of Ibuprofen loaded niosome formulation (F9).

Based on various parameters, the F_9_ gave optimum results and found to be the most suitable formulations that further subjected to various release kinetic models such as zero-order kinetics, first-order kinetics, Higuchi’s model kinetics, Korsemeyer’sPeppas model kinetics, and Hixson-Crowell model kinetics. The results were graphically shown in Figure 7. The formulation (F_9_) best followed zero-order release kinetics with a fickian release mechanism. The kinetic analysis of all release profiles followed Fickian diffusion-controlled mechanism followed by a slower zero-order release. The drug release in the slower phase was regulated by diffusion through the swollen niosomal bilayers (60). 

*Selection of Best Batch*

All the formulations were optimized based on yield%, Vesicle size, drug% entrapment efficiency, and *in-vitro* release in 8 h duration. Based on the results, formulation F_9_ was supposed to exhibit optimum results within the desired range. Hence, this formulation was subjected to further incorporation into the gel system.

*Evaluation of Ibuprofen loaded Niosomal Gel*

All the three Ibuprofen niosomes loaded gel formulations were prepared and further introduced for different evaluation parameters.

*Ex-vivo diffusion studies*

**Figure 8.**
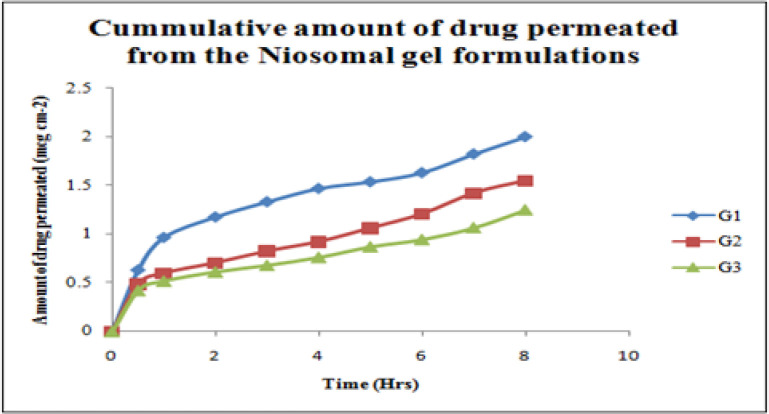
Cumulative amount of Drug Permeation from all Ibuprofen loaded niosomal gel system (G1, G2, G3).

The selected gels were evaluated for *in-vitro* skin permeation studies. *In-vitro *drug release of niosomal gel formulations were observed up to 8 h and showed a slow release pattern in all of the three niosomal gel formulations as shown in Table 4. An inverse relationship was observed between the permeation rate and the viscosity of the gel formulation. The data is graphically represented in Figure 8.

*In-vivo anti-inflammatory activity*

**Figure 9:**
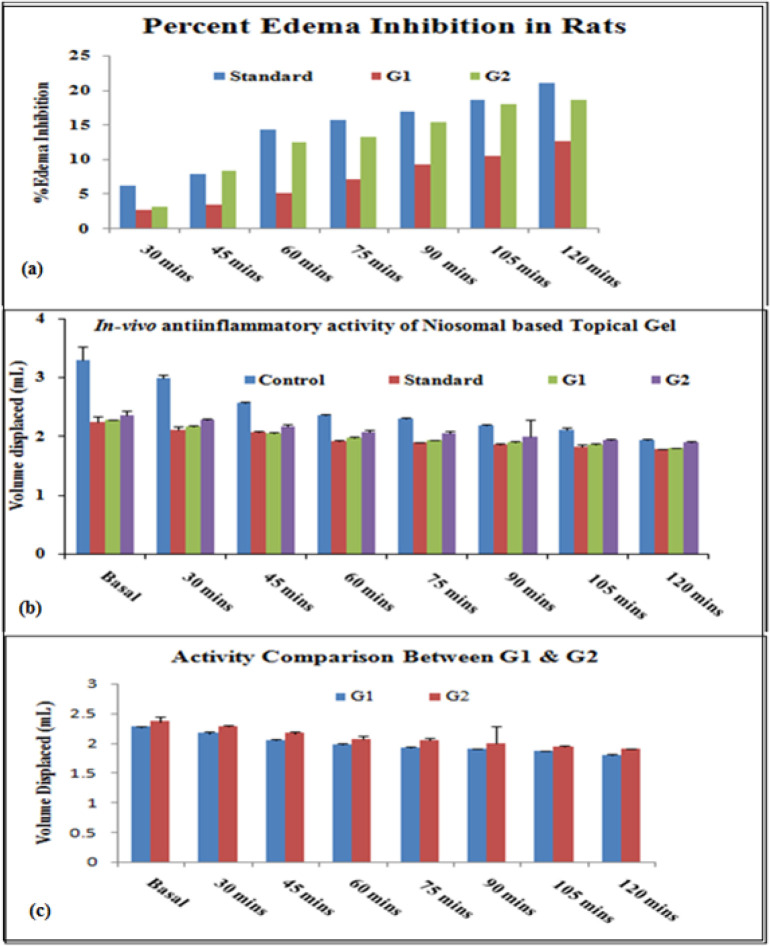
Comparison plots representing in-vivo studies: Percent edema Inhibition and anti-inflammatory activity in animal groups for Ibuprofen loaded niosomal gel system (G1 and G2).

*In-vivo* anti-inflammatory activity was performed using the “Carrageenan induced rat paw edema” model. Percentage inhibition of inflammation in control and treated groups was calculated for different formulations (Standard, G_1_, and G_2_) and results were shown in Figure 9 and Table 5. Percentedema inhibition was found maximum in both the niosomal gel formulations (G_1_ and G_2_) as compared to the standard (Ibuprofen marketed plain gel) up to 2 h. Data significance was calculated for all formulations (standard, and test (G_1_ and G_2_)) to control groups and the test formulations (G_1_ and G_2_) were also compared. 

*Skin-irritancy study*

Based on the results obtained from the above studies, it has been found that formulation G2 showed optimum results within the desired range. Hence, formulation G2 was subjected to skin irritancy study on animals (albino rat). Following 3 days application of the niosome loaded gel; the results of the skin irritation test indicated that the G2 gel did not cause any skin reaction. It can be assured that Ibuprofen loaded noisome loaded gel did not cause any skin irritation and can be used as topical gel formulation.

## Discussion

The average sizes of niosomes increased when the HLB values of the surfactant decreased from Tween to Span as the diameter of the vesicles is dependent on the length of the alkyl chain of the surfactant and may contribute the larger vesicle size (61, 62). The high drug entrapment with Span 20 and Tween 80 may be attributed to the fact that surfactants of higher “Transition temperature” (Tc) which are present in the ordered gel form forming less leaky bilayers as compared to surfactants of lower Tc. On the other hand, a high degree of hydration of the hydrophilic head group and its longer alkyl chain (C18) of Tween 80 in water may also result in high drug entrapment which supports Tween based niosomes formulations in respect to sphericity, entrapment, and chemical stability (63- 65). From the phase-contrast study, the best niosome formulation was selected based on sphericity among all niosome formulations and the selected formulation (F9) was further evaluated by higher resolution technique *i.e.* SEM analysis. The formulation F9 was found to be spherical. The surfaces were smooth without forming any agglomerations and also showed surface adhered drug vesicle. However, fatty alcohol debris is seen around the surface of a single Ibuprofen loaded niosome structure as revealed by SEM images which could be due to the unconsumed cetosteryl alcohol by the niosomes bilayers during the niosomes preparation. From zeta potential analysis, it can be said that cetosteryl alcohol which is located within the hydrophobic tails of Tween 80 in the vesicles may tighten the bilayer membranes and thus the formulation exhibited negative charge. It is also well established that the large size vesicles in the formulation acquire more negative charge (66). The presence of a hydrophilic surfactant in the bilayer structure has been reported to form unilamellar vesicles. The reported research explained that the system having the ZETA Potential values positive more than +30 or more negative than -30 mV remains stable thus the stability of vesicles may contribute to sustaining the release of drug in the system at a controlled rate (67). The *in-vitro *release profiles of Ibuprofen from Niosomes showed the CPR of all niosomal vesicles ranged between 32.08 ± 0.43% to 50.50 ± 0.60% after 8 h which indicated the modified release of the Ibuprofen for a longer duration. Significant changes in release upon changing the type of surfactant in the niosome preparations. Comparing the release profile of formulation Span 60 (4:1) and Tween 80 (2:1) with other formulations; higher release rate and efficiency were observed. The fact may be that using surfactants with high HLB value supposes to have higher solubilizing power on hydrophobic solutes in an aqueous medium which may reveal a significant increase in the drug release when compared with hydrophobic surfactants (68, 69). The formulation (F9) shows the best correlation coefficient (R2) value *i.e* 0.988, thus followed zero-order release kinetics with Fickian diffusion-controlled mechanism (60). After optimizing niosomes formulations; the best formulation was transferred into the gel system. All the formulations were shear thinning and exhibited pseudoplastic behavior. The flow behavior of the formulations was shown to be non-Newtonian pseudoplastic flow and thus said to have good rheological properties. The spreadability of formulated gels was decreased as the concentration of polymer increased. The values of spreadability indicate that the gel is easily spreadable by the small amount of shear. *Ex-vivo* diffusion study showed a linear relationship between the cumulative amount permeated and time, indicating zero-order permeation kinetics and the permeation of Ibuprofen was based on diffusion-controlled mechanism. The significantly low flux values of Ibuprofen were found in gels G_2_ and G_3_ as compared to that of niosomes loaded carbopol gel G_1_. The results showed decrease in drug permeation and drug flux values with increased concentration of *Lallemantia royaleana Benth. *thatsuggestsmucilage have a good binding to the system and therefore controlled the release of Ibuprofen from the niosomal gel system (70). The *in-vivo* results of test formulations (G1 and G2) indicated high skin retention and enhanced penetration rates within the skin. According to both *ex-vivo* and *in-vivo* studies G2 niosomal gel formulation showed better permeation and effectiveness as compared to G1 niosomal gel formulation. This probable reason may be due to higher skin retention and deposition of the G2 niosomal gel formulation resulting in higher partitioning of the drug into the rat paw which may be responsible for its prolonged and enhanced anti-inflammatory activity (71). Thus, formulation (G_2_) may help in improving the therapeutic index and is also expected to minimize the side effects due to selective build-up of drug concentration at the site of action (72). Skin irritancy results indicated that the G2 gel did not cause any skin reaction and can be used as gel formulation.

**Table 1:** Formulations (Surfactant: Cholesterol Ratio), Entrapment efficiencies (EE%), Mean Vesicle Size (MVS), Cumulative Percent Release (CPR) of all Ibuprofen loaded Niosome Formulations

**Formulations Code**	**MVS ± SD (μm)**	**EE ± SD (%)**	**CPR ± SD**
F_1 _(Span 20: Chol, 2:1)	3.8 ± 0.66	52 ± 1.0	45.2 ± 0.93
F_2 _(Span 20: Chol, 4:1)	4.1 ± 0.89	34 ± 1.53	45.1 ± 1.54
F_3 _(Span 40: Chol, 2:1)	4.3 ± 1.12	44 ± 1.0	43.1 ± 0.75
F_4 _(Span 40: Chol, 4:1)	4.7 ± 1.04	36 ± 1.53	38.1 ± 0.43
F_5 _(Span 60: Chol, 2:1)	4.5 ± 0.47	35 ± 1.73	32.8 ± 0.43
F_6 _(Span 60: Chol, 4:1)	5.2 ± 0.92	35 ± 1.0	50.5 ± 0.60
F_7 _(Tween 60: Chol, 2:1)	3.6 ± 1.33	37 ± 1.0	46.9 ± 0.39
F_8 _(Tween 60: Chol, 4:1)	4.1 ± 1.21	42 ± 1.53	40.8 ± 0.55
F_9 _(Tween 80: Chol, 2:1)	3.2 ± 0.75	51 ± 1.0	50.2 ± 1.18
F_10 _(Tween 80: Chol, 4:1)	4.1 ± 0.82	46 ± 1.0	45.5 ± 1.2

Results were presented as mean standard deviation (n = 3).

**Table 2:** Formulation table for preparation of niosomal gel

**Code**	**Selected Niosome formulation**	***Lallemantia royaleana Benth.***	**Carbopol-934**
G1	F9	-	2.0%
G2	F9	1.0%	1.0%
G3	F9	1.5%	1.0%

Glycerin (10%), Benzalkonium chloride (0.01%), Propylene glycol (5%), Triethanolamine (0.1%) were used for each formulation.

**Table 3:** Various animal groups used for *in-vivo* anti-inflammatory study

**Groups**	**No of animals**	**Description**
1	6	Served as normal or Untreated control group
2	6	Received standard Ibuprofen topical Gel (Ibugel) equivalent to 720 µg/100 g (25 mg of gel) of the Ibuprofen topically
3	6	Received Ibuprofen loaded niosomal gel formulation (2% Carbopol) equivalent to 720 µg/100 g (25 mg of gel) of the Ibuprofen topically which served as G_1_
4	6	Received Ibuprofen loaded niosomal gel formulation (1% Balanga + 1% Carbopol) equivalent to 720 µg/100 g (25 mg of gel) of the Ibuprofen topically which served as G_2_

**Table 4:** Results for Amount permeated, Drug flux, and Log permeability coefficient of the Ibuprofen loaded Niosomal gel formulations

**Gel Formulations**	**Amount of drug permeated[Q] (mg cm** ^-2^ **) ± SD**	**Drug flux [Jss] (mg cm** ^-2^ ** h** ^-1^ **) ± SD**	**Log Permeability Coefficient (Log Kp) ± SD**
**G1**			
(2% Carbopol)	1.999 ± 1.34	10.66 ± 1.82	0.546 ± 0.58
**G2**			
(1% *Lallemantia royaleana Benth.*+1%Carbopol)	1.548 ± 1.09	8.256 ± 1.15	0.433 ± 0.76
**G3**			
(1.5% *Lallemantia royaleana Benth.*+1% carbopol)	1.248 ±1. 27	6.657 ± 0.91	0.339 ± 0.93

Results were presented as mean standard deviation (n = 3).

**Table 5:** Percentage inhibition of the edema in albino rats

**Time (min)**	**Control (%)**	**Standard (%)**	**G**_1_ ** (%)**	**G**_2_ ** (%)**
Basal	-	0	0	0
30	-	6.2	2.8	3.17
45	-	8.0	3.5	8.46
60	-	14.4	5.2	12.5
75	-	15.7	7.16	13.32
90	-	16.89	9.3	15.43
105	-	18.67	10.5	17.97
120	-	21.0	12.7	18.66

## Conclusion

The present work was a satisfactory preliminary study in developing the niosomal gel system of Ibuprofen. Ibuprofen is a non-steroidal anti-inflammatory agent, used in the treatment of inflammation, and was successfully loaded into the niosomal gel system. Theprepared formulations were evaluated for yield values, vesicle size, entrapment efficiencies, and *in-vitro* release studies. The developed formulation G_2_ is a viable alternative to conventional topical gels as it provides sustained delivery. Moreover, the natural mucilage, used in the gel, possess anti-inflammatory properties suggesting to exert a synergistic action of niosomal gel system at the target site to improve overall effect. Thus, it can be concluded that the niosomes loaded gel can be a promising potential drug delivery system for topical application to efficiently target local pains for prolonged periods and to reduce the frequency of application of gels as compared to conventional gels.
